# Standard Bacteriophage Purification Procedures Cause Loss in Numbers and Activity

**DOI:** 10.3390/v13020328

**Published:** 2021-02-20

**Authors:** Amanda Carroll-Portillo, Cristina N. Coffman, Matthew G. Varga, Joe Alcock, Sudha B. Singh, Henry C. Lin

**Affiliations:** 1 Division of Gastroenterology and Hepatology, Department of Internal Medicine, University of New Mexico, Albuquerque, NM 87131, USA; ACarrollPortillo@salud.unm.edu; 2 Biomedical Research Institute of New Mexico, Albuquerque, NM 87108, USA; Cristina.Coffman2@va.gov (C.N.C.); MatthewGVarga@gmail.com (M.G.V.); Sudha.Singh@va.gov (S.B.S.); 3 Department of Emergency Medicine, School of Medicine, University of New Mexico, Albuquerque, NM 87131, USA; JoAlcock@salud.unm.edu; 4 Medicine Service, New Mexico VA Health Care System, Albuquerque, NM 87108, USA

**Keywords:** bacteriophage, purification, CsCl, PEG precipitation, M13, T4, ΦX 174

## Abstract

For decades, bacteriophage purification has followed structured protocols focused on generating high concentrations of phage in manageable volumes. As research moves toward understanding complex phage populations, purification needs have shifted to maximize the amount of phage while maintaining diversity and activity. The effects of standard phage purification procedures such as polyethylene glycol (PEG) precipitation and cesium chloride (CsCl) density gradients on both diversity and activity of a phage population are not known. We have examined the effects of PEG precipitation and CsCl density gradients on a number of known phage (M13, T4, and ΦX 174) of varying structure and size, individually and as mixed sample. Measurement of phage numbers and activity throughout the purification process was performed. We demonstrate that these methods, used routinely to generate “pure” phage samples, are in fact detrimental to retention of phage number and activity; even more so in mixed phage samples. As such, minimal amounts of processing are recommended to introduce less bias and maintain more of a phage population.

## 1. Introduction

Bacteriophage, viruses that prey on bacteria, were discovered in the 1900s independently by Félix d’Hérelle and Frederick Twort. The potential for phage to be used in bacterial infections was recognized soon after their discovery, ushering in decades of research into phage therapy [1,2]. With the advent of antibiotics, phage research continued but with a focus more on molecular genetics and mechanisms. Now that antibiotic resistance is on the rise, phage has once again come to the forefront as a potential therapeutic strategy. However, beyond recognition of the advantages particular phages present for therapeutic reasons, there is an emphatic need for research into the role of phage as a key component and regulator of the microbiome [3,4]. Of particular interest is the mixed phage community that is part of the gastrointestinal microbiome, specifically for its role in both regulation of the bacterial populations and activation of the metazoan immune system [5,6,7]. Better understanding of the dynamics of the phage population as a part of the microbiome offers promise for novel therapeutic strategies for treating gastrointestinal dysbiosis expanding upon the traditional definition of phage therapy. Successful purification of the entire mixed community while maintaining activity and numbers is necessary in order for research on phage dynamics within the gastrointestinal system to progress. Additionally, purification procedures that maximize intact, active phages while minimizing cost and complexity are desirable.

There are several well-described methods for purification of samples containing bacteriophage, both obtained from bacterial cultures and from environmental samples [8,9,10,11,12,13,14,15]. Filtration, polyethylene glycol (PEG) precipitation, and cesium chloride (CsCl) gradient centrifugation are among the most frequently used techniques as they are simple, low-cost, well-characterized methods. However, these particular purification procedures themselves have been observed to negatively affect some phage [10,15], a factor typically outweighed by the need for sample concentration (PEG precipitation) and removal of impurities such as LPS or bacterial detritus (CsCl gradient purification). When dealing with environmental phage populations, one must consider the benefits of concentration and purification in relation to accurate preservation of phage diversity and activity. To mitigate losses to phage integrity caused by the standard purification protocols, new separation methods have been described including tangential flow filtration (TFF) [8,16] and liquid chromatography [1,17]. While it is possible that these new methods will prove to be more advantageous to maintaining population integrity for environmental samples, at this time they remain untested against a variety of phage and are difficult for many laboratories to set up easily.

Ultimately, when considering purification approaches for any given phage containing sample, end purpose is critical. PEG and CsCl purification procedures are prolific in the literature for purification of individual phage, where starting volumes/concentrations are determined by amount of bacterial culture. In these instances, phage characterization is often not compromised by loss of phage number or activity during purification as, despite losses, there is still ample quantity of viable phage to carry out evaluations of phage function. In contrast, purification of phage populations from environmental samples often requires precipitation to concentrate phages into a manageable volume for experimental use and gradient purification to remove impurities that may affect downstream applications. In these instances, loss of phage number and viability are more detrimental to follow on evaluations. 

With this in mind, we find that most protocols discussing purification of phage populations have been done for the purpose of preparing samples for metagenomic sequencing to examine the phage constituents of a community [9]. The PEG and CsCl gradient purification preparatory steps have been applied widely without questioning. Studies using these purification steps have generated data that have been used to understand much about the bacteriophage that reside within the microbiome and how their populations fluctuate in relation to any number of conditions. While optimization of these protocols has been performed to maximize the amount of DNA purified from fecal samples [9], examination of the physiological role of phage populations in different conditions requires understanding the effects of purification processes on overall phage number, activity and population dynamic. Previous examination of the effects of purification steps on phage viability have relied on spiking of environmental samples with known phage, usually from the order Caudovirales. While informative for examples of what may effect tailed phages, little is known about the effect of these methods on the numbers and activity of a mixed phage population. Given that there are a variety of phage beyond Caudovirales that are common to a healthy human gastrointestinal tract [18,19,20,21,22], it is necessary to understand how the most common purification strategies may influence phage population outcomes. In this study, we tested the hypothesis that PEG and CsCl purification strategies, the most likely to be employed, may result in a significant loss of phage number and activity by examining the retention of phage number and activity of three known phages from different families of varying size, shape, and density and their mix following PEG precipitation and CsCl gradient purification.

## 2. Materials and Methods

### 2.1. Bacteria and Bacteriophage

**Figure 1:**
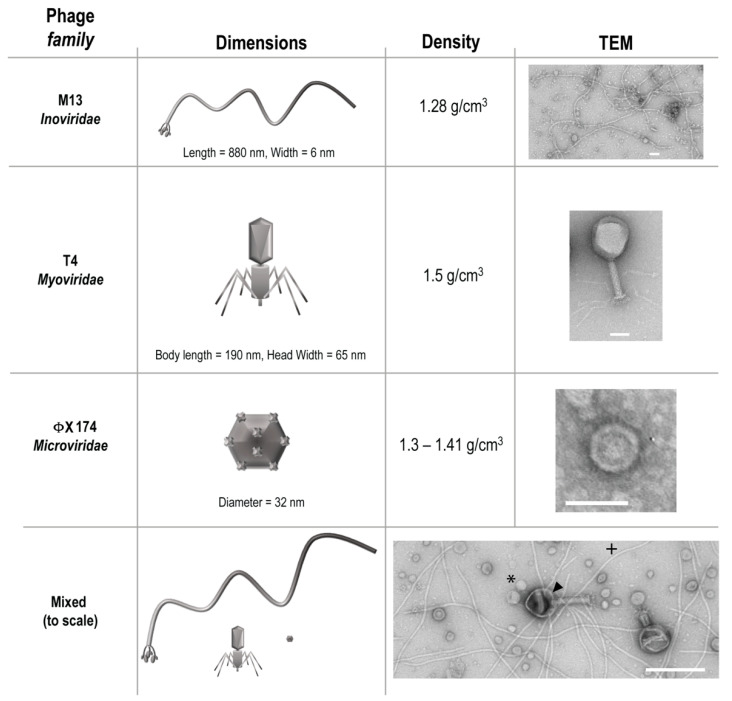
Characteristics of bacteriophage used. Arrowhead = T4, * = ΦX 174, **+** = M13.

Three phages from different families, T4, M13, and ΦX 174 were purchased from ATCC (Manassas, VA, USA) along with the *Escherichia coli* host strains K803 (for T4) and strain C (for ΦX 174). *E. coli* host strain DH5**α** F’Iq (for M13) was purchased from New England Biolabs (Ipswich, MA, USA). These phages were chosen to represent bacteriophage diversity of size, structure, and density (Figure 1), a heterogeneity that would be natural in a gastrointestinal environment. 

### 2.2. Growth and Isolation of Phage

Host bacterial strains were grown to mid-log phase in Luria Bertani broth (MilliporeSigma, Burlington, MA, USA) at 37 °C with shaking, and high titer phage (10^8^–10^10^) was added at a 1:10 dilution (by volume). ΦX 174 (ΦX) and T4 were propagated for approximately 5 h until cultures were cleared. M13 cultures were incubated overnight. To stimulate release of lysogenic phage, bacteria were treated with 8–15 μg/mL mitomycin C (MMC, MilliporeSigma, Burlington, MA, USA) overnight at 37 °C. At collection, bacteria were pelleted from phage cultures with two rounds of centrifugation at 10,000× *g* for 30 min at 4 °C followed with filtration of the clarified supernatant twice through 0.45 μm pore filters. Phage were stored at 4 °C and used within the month.

### 2.3. PEG Precipitation

Twenty mL of each filtered phage supernatant was treated with 10% (*w*/*v*) PEG 6000, 8000, or 10,000 (MilliporeSigma, Burlington, MA, USA) overnight at 4 °C to allow for precipitation of phage. Some protocols use additional NaCl to enhance precipitation [18,19,20,21,22]. We found that addition of NaCl to our samples resulted in loss of pellet, so it was not utilized in final preparations. The following day, samples were pelleted at 4500× *g* for 30 min at 4 °C and supernatants were gently poured off without disturbing the pellet. Pellets were then resuspended in 5 mL of SM Buffer (100 mM NaCl, 8 mM MgSO_4_·7H_2_O, 50 mM Tris-Cl, pH = 7.5). One mL of sample was removed for planned assays and stored at 4 °C until use (within a month).

### 2.4. CsCl Gradient Centrifugation

Four mL of each PEG precipitated sample was loaded onto a CsCl step gradient with densities of 1.4, 1.5, and 1.6. Samples were centrifuged in a SW41Ti rotor (Beckman, Indianapolis, IN, USA) at 100,000× *g* for 2 h at 4 °C. After centrifugation, banded material was removed from the gradient (consistently ≤1.5 mL) and put into dialysis cassettes with a 3K MWCO (ThermoFisher, Waltham, MA, USA). Samples were dialyzed against 1000× volume of SM buffer at 4 °C for 3 h with one buffer change. After dialysis, samples were brought up to a 4 mL volume with sterile SM buffer and filtered once more with a 0.45 μm syringe filter (MilliporeSigma, Burlington, MA, USA).

### 2.5. Epifluorescent Imaging of Phage DNA

Using a glass vacuum filtration set up, phage DNA for each sample set and the associated stock solution were applied to filters for staining and visualization with epifluorescent microscopy. Whatman Anodisc 25 mm diameter supported membrane 0.02 μm filters (Tisch Scientific, North Bend, OH, USA) were applied to the support surface of the vacuum filtration device. As previously described [23], 100 μL of phage treatment (PEG precipitated or CsCl purified) or 400 μL of phage stock (untreated, concentration equivalent) were diluted in 5 mL SM buffer and the entire volume was applied to the filter via vacuum. Filters were air dried and then laid sample side up onto a 50 μL drop of 400× Sybr Green (ThermoFisher, Waltham, MA, USA) and incubated for 20 min to stain nucleic acid. Filters were washed three times by laying them on three subsequent drops of 100 μL of sterile PBS before wicking excess liquid and laying them sample side up on a glass slide. ProLong Gold (ThermoFisher, Waltham, MA, USA) was applied to mount the filter on to a glass coverslip and slides were cured overnight at room temperature to allow for hardening. Epifluorescent imaging of filters was performed on a Fluoview Olympus microscope (Olympus Life Science, Center Valley, PA, USA) using a 63× oil immersion objective. Visualization was achieved with a 488 laser and application of a fully open pinhole. Ten fields of view were imaged per filter with imaging parameters remaining the same for all samples in the same phage set to allow for comparison of fluorescent labeling. Images were processed and analyzed using FIJI software (ImageJ, [24]). Measurements were input into GraphPad Prism software (GraphPad, San Diego, CA, USA) for final graphing and statistical analyses. 

### 2.6. Detection of Phage Activity

Phage overlay assays were performed as described [25] to characterize phage activity as a function of each treatment during purification. Host bacteria were grown overnight in LB broth. LB sloppy agar (0.6% agar) was heated to melting, aliquoted into tubes at a 7 mL volume, and maintained at 45 °C. One mL of bacterial host growth and 100 μL of phage dilution were added to a sloppy agar aliquot and poured immediately onto a solid LB agar plate as an overlay with gentle swirling to ensure even distribution. Solidified plates were placed in a 37 °C incubator for overnight incubation. Resulting phage plaques were counted and used to calculate plaque forming units (PFU) within the original phage sample. Prism software was used to graph and analyze data.

### 2.7. Isolation of Phage DNA and qPCR

To quantitate phage numbers in samples, phage DNA was isolated using a phenol chloroform extraction method as previously described [26], and qPCR with phage specific primers was performed. Briefly, 500 μL (M13 and mixed, PEG and CsCl) or 2 mL (T4, ΦX 174, and mixed, CsCl) of phage samples were treated with DNase I (final concentration 10 μg/mL) at 37 °C for 30 min. After DNase I inactivation at 65 °C for 10 min, SDS (final concentration 0.5%) and Proteinase K (final concentration 0.1 mg/mL) were added for 30 min at 37 °C. Samples were treated with an equal volume phenol:chloroform:isopropanyl (p:c:i, 25:24:1, MilliporeSigma, Burlington, MA, USA) and centrifuged for 5 min at 1500× *g*. The top phase was removed to a new tube and the p:c:i step was repeated. The top phase was then treated with an equal volume chloroform:isoamyl alcohol (24:1, MilliporeSigma, Burlington, MA, USA) and centrifuged for 5 min at 6000× *g*. The top phase was moved to a new tube, and DNA was precipitated with Sodium acetate (final concentration 0.3M, MilliporeSigma, Burlington, MA, USA) and equal volume 100% isopropanol (MilliporeSigma, Burlington, MA, USA). DNA was pelleted at 20,000× *g* for 30 min at 4 °C and pellets were washed with 500 μL ice cold ethanol twice with pelleting at 20,000× *g* for 5 min at 4 °C after each wash. Pellets were air dried prior to resuspension in 50 or 100 μL of water. qPCR with purified phage DNA was performed using primers (IDT) specific for ΦX 174 (forward: TTACTGAACAATCCGTACGTTTCCA reverse: ACGGCAGAAGCCTGAATGAG), T4 (forward: ACTGGCCAGGTATTCGCA, reverse: ATGCTTCTTTAGCACCGGCA), or M13 (forward: CACCGTTCATCTGTCCTCTTT, reverse: CGACCTGCTCCATGTTACTTAG) with Luna Master Mix (New England Biolabs, Ipswich, MA, USA). Forty cycles were performed with a 55 °C annealing temperature and 30 s extension time. 

### 2.8. Transmission Electron Microscopy (TEM)

Four hundred-mesh Cu grids with carbon films (made in-house) were glow discharged for 30 s (Harrick PDC-32G Plasma Cleaner; Harrick Plasma, Ithaca, NY, USA). For sample prep, grids were floated face-down on a 10 μL droplet of sample for 10 min at room temperature, and then rinsed through 3 droplets (20 μL each) of deionized water. Excess liquid was wicked off with a filter paper triangle and grids were incubated for 2 min on 20 μL droplets of 1% uranyl acetate. Excess liquid was again wicked off and grids were left on filter paper to air dry. Grids were imaged at 80 kV in a Hitachi HT7700 TEM and images were collected with an AMT XR16M 16-megapixel digital camera.

### 2.9. Phage Preparation Workflow

**Figure 2:**
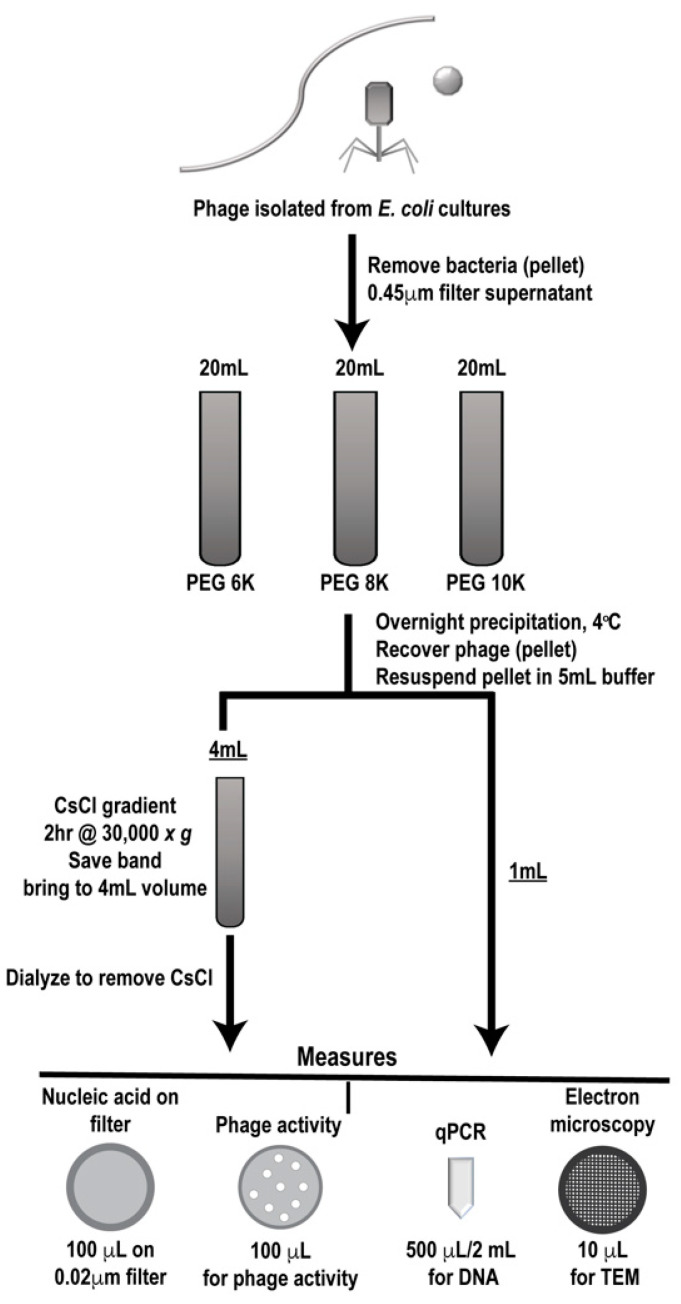
Work flow for phage preparation. Outline of phage preparation procedure showing where collection points occurred, purification steps taken (polyethylene glycol (PEG) precipitation followed by cesium chloride (CsCl) gradient purification) and types of analyses used (measures).

Phage were grown and isolated as described and clarified phages were either individually processed or mixed in equal volumes through a designated workflow (Figure 2). Samples were first PEG precipitated followed by CsCl gradient purification, two commonly employed techniques for phage enrichment. In the literature, different molecular weight PEG has been used for precipitating various individual phages [27,28,29,30,31], so we utilized three different PEG molecular weights (6K, 8K, and 10K) for precipitation to determine if this variable affected phage population during purification. Processing of each sample set (individual and mixed) was performed in triplicate to provide statistically relevant analyses. Prior to and after each processing step, samples were analyzed for phage activity (phage overlay assay, PFU), phage number (qPCR and epifluorescence), and structure (TEM) and results were compared to the values expected as determined from the stock phage solutions. In the figures, samples collected after PEG precipitation are denoted as such, “PEG”, along with the molecular weight of the PEG used. Likewise, samples that were collected after CsCl gradient purification are labeled “CsCl” along with the molecular weight of the PEG used for the original precipitation (6K, 8K, or 10K).

## 3. Results

### 3.1. TEM Shows Concentration of Phage Samples by Both PEG and CsCl Gradient

**Figure 3:**
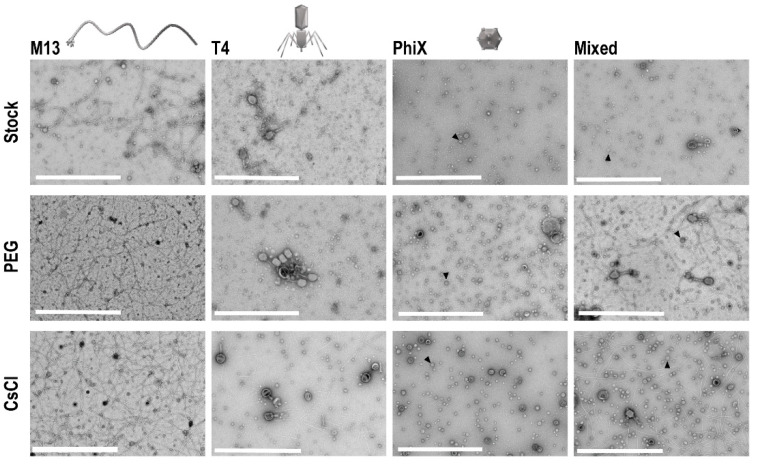
TEM of phage samples. Focused regions of selected images from each set of phage samples to demonstrate enhancement of phage populations after PEG concentration and CsCl gradient purification. An example ΦX phage particle is denoted with an arrowhead in each sample frame where they are found. Scale bars are 1 μm.

To determine if concentration of phages occurred with PEG precipitation and CsCl gradient purification, and to assess overall phage structure post-processing, TEM was performed on all phage samples. Ten microliters from each sample and the respective stock phage solutions were processed on separate TEM grids for imaging. Five images of each grid were taken at random to allow for assessment of the structure and general phage number present in each sample. In each instance, grids for processed samples (i.e., PEG and CsCl gradients) possessed more phage as compared to grids with stock phage solutions (Figure 3). Structurally speaking, phages remained consistent throughout the purification procedures except for T4 phages, which visibly stained differently after CsCl gradient purification (darker staining of phage heads). 

### 3.2. PEG Precipitation and CsCl Gradient Purification Result in Loss of Phage Activity

**Figure 4:**
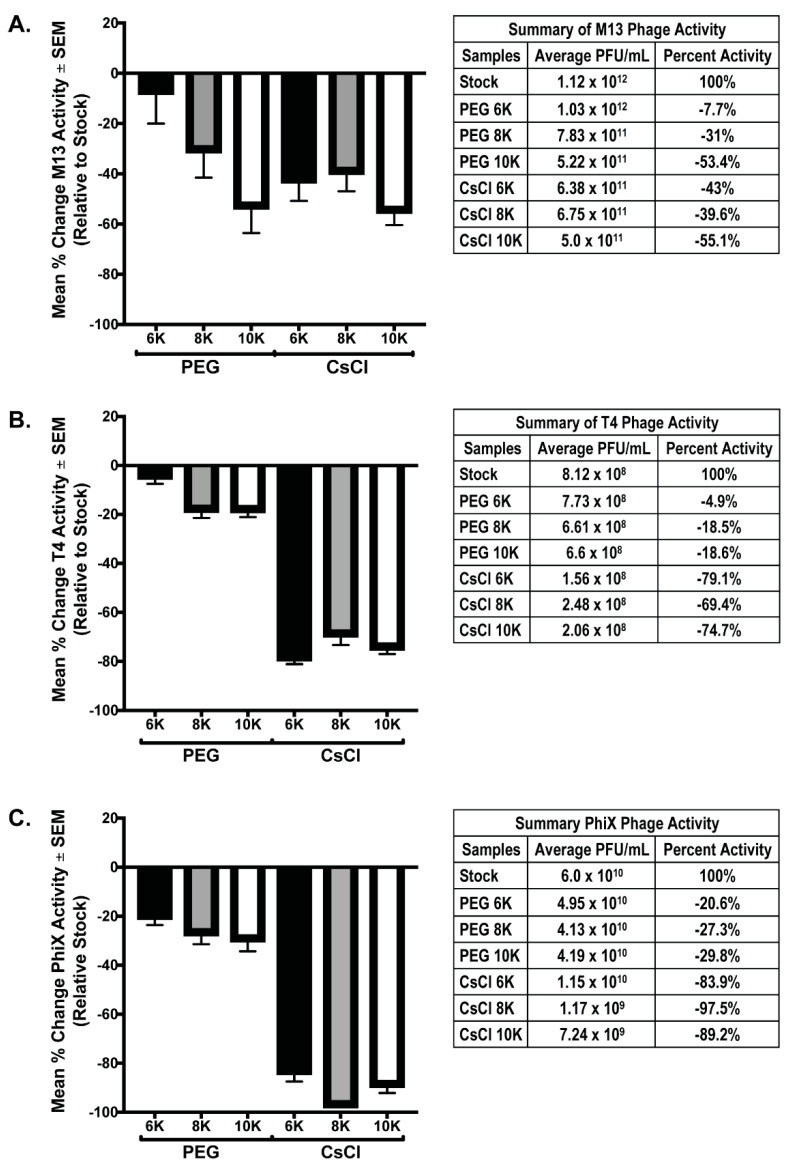
Phage activity decreases after PEG and/or CsCl purification. Graphical representation of the percent difference between the detected number of plaque forming units (active phage) and the expected number calculated from stock phage solutions for each individual phage preparation: (**A**) M13, (**B**) T4, and (**C**) ΦX. Tables accompanying each graph list numerical values for the respective bar and the stock phage activity value from which calculations were based.

Both PEG precipitation and CsCl gradient purification are frequently used in phage purification protocols to help concentrate and purify phage populations for downstream procedures. However, these treatments have been found to be detrimental to phages [8,27]. As such, we performed phage overlay assays on all phage samples prior to and after purification steps to determine the effects of each of these procedures on overall phage activity. For each individual phage preparation, activity after PEG and CsCl gradient purification was determined using a phage overlay assay. PFU/mL for each sample was calculated and compared to the expected value determined from the PFU/mL value of the starting stock phage solutions; the mean percent change of each sample relative to expected (set to zero) was graphed (Figure 4). Testing phage activity of the mixed samples was not possible as all phage used in these experiments are coliphage, and as such, will form plaques that cannot be differentiated by phage type. We hypothesized that if concentration was successful without loss of activity, the measured activity should exceed that of the stock phage solution by 4-fold due to concentration occurring during the PEG precipitation. Additionally, activity should remain the same after PEG and CsCl gradients. Instead, the results demonstrate that both PEG precipitation and CsCl gradient centrifugation cause substantial loss of phage activity (denoted by a negative mean % value). In all phage tested, precipitation with PEG 6K was the least detrimental to phage activity (M13 = −7.7% ± 12.3; T4 = −4.9% ± 2.6; ΦX = −20.6% ± 3.0). The degree to which purification of phage affected the activity varied with each phage tested. Of all phage tested, M13 activity showed the least variation between samples with significance in two-way ANOVA of *p* = 0.023. Multiple comparisons showed that the only M13 samples with significant difference were the PEG 6K (average = 1.3 × 10^12^) and PEG 10K (average = 5.2 × 10^11^), *p* = 0.022. In contrast, activity loss for both T4 and ΦX samples was significant in two-way ANOVA (*p* < 0.0001) with multiple comparisons, showing significant loss occurring between the PEG precipitation and CsCl gradient purification steps (*p* < 0.0001). Similar to M13 results, there was significance (*p* = 0.039) between the T4 PEG 6K (average = 7.73 × 10^8^) and T4 PEG 10K (average = 6.6 × 10^8^) samples. These are the only indication of negative effect on phage activity due to a difference in the molecular weight of PEG used for precipitation, although this effect was not evident in the ΦX samples. It is important to note that while the graphs demonstrate that a large loss in phage activity occurred as purification continued, even samples with the most dramatic losses still possessed a substantial amount of phage activity, and by connection, phage number (Figure 4, tables). For example, there was an almost complete loss of activity from what is expected in the ΦX CsCl 8K sample, however, there was still an average of 1.17 × 10^9^ PFU from these samples in an overlay assay indicating, at minimum, an equivalent number of phage/mL still within the sample. 

### 3.3. CsCl Gradient Purification Results in Loss of Phage Numbers

**Figure 5:**
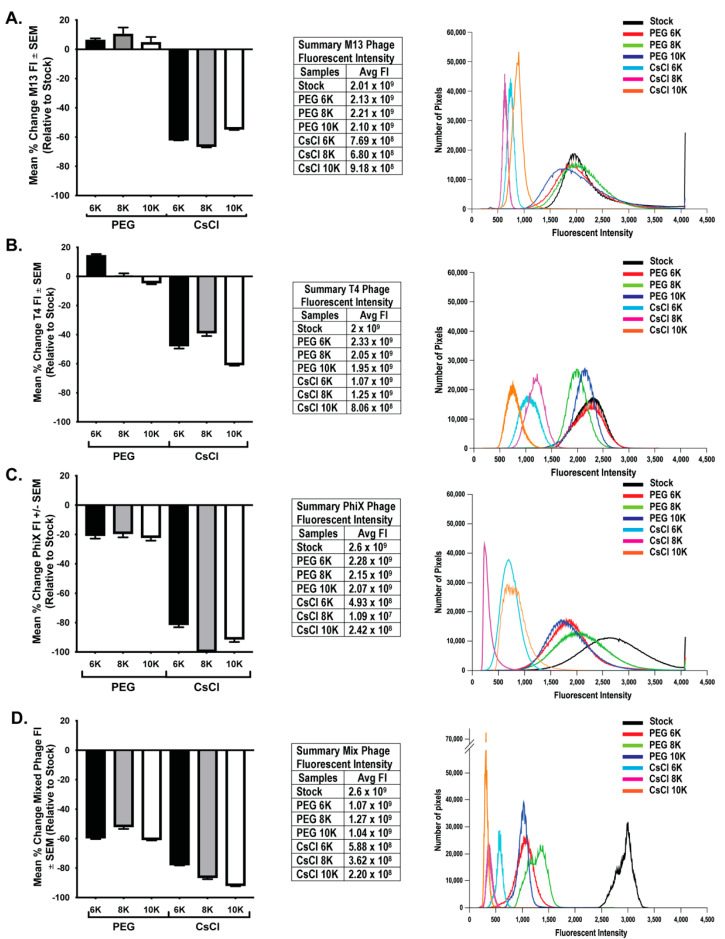
Greater loss of phage nucleic acid after CsCl purification than PEG precipitation. Graphical representation (left panels) of the mean percent change in nucleic acid fluorescent intensity (FI) relative to expected levels after treatments for (**A**) M13, (**B**) T4, (**C**) ΦX, and (**D**) mixed phages. The fluorescent intensity averages for each sample as well as the expected levels (stock) are listed (middle panels) and an overlay of representative histograms for each phage from all samples tested (right panels) show shifts in fluorescent intensities from expected (black lines, stock) of nucleic acid labeling due to treatments.

To determine if loss of phage activity during purification correlated with an overall loss in phage number, we performed epifluorescent microscopy on filters with labeled phage as previously described [23]. This method has been used to quantify number of phages in a sample [9,32] by counting the number of punctate spots (indicative of phage) in a known area of the filter. However, one cannot easily ascertain the true number of phage particles in any given pixel/spot. This is particularly true of smaller phage particles such as ΦX 174, which could potentially have tens to hundreds of phage particles exist within a single pixel space. Because of these considerations, an average total fluorescent intensity (FI) was calculated from 10 fields of view from each sample filter. As PEG precipitation results in a four-fold concentration of sample, control filters were generated using an equivalent volume of stock to allow for direct comparison of qualitative changes in the amount of phage nucleic acid due to purification procedures. FI values were background subtracted and normalized to expected levels calculated from stock FI values. Similar to the presentation of phage activity data, the mean percent change of FI for each individual phage and mixed phage sample were graphed (Figure 5, bar graphs) and the average FI values are presented in table format (Figure 5, tables). Further demonstration of the general fluorescent trend within each set of phage data is presented in histogram format with overlaid histograms from individual representative fluorescent images for each treatment (Figure 5, histograms). Results from individual and mixed phage samples showed a consistent trend in that CsCl gradient purification resulted in significant loss of nucleic acid (two-way ANOVA of PEG and CsCl samples; M13 *p* < 0.0001, T4 *p* < 0.0001, ΦX *p* < 0.0001, Mixed *p* < 0.0001). The effect of PEG molecular weight differed between phage. With M13, there was only small significance (*p* = 0.039) between CsCl 8K (average FI = 6.8 × 10^8^) and CsCl 10K (average FI = 9.18 × 10^8^). With T4, there was significant difference (*p* < 0.001) between PEG 6K samples (average = FI 2.33 × 10^9^) and PEG 8K (average 2.05 × 10^9^) or PEG 10K (average 1.95 × 10^9^) samples. All T4 CsCl results were significantly different: CsCl 6K (average = 1.07 × 10^9^) vs. CsCl 8K (average = 1.25 × 10^9^) (*p* = 0.028) or CsCl 10K (average = 8.06 × 10^8^) (*p* < 0.0001) and CsCl 8K vs. CsCl 10K (*p* < 0.0001). With ΦX, PEG samples were not statistically different, but CsCl 6K (average = 4.93 × 10^8^) was significantly different from CsCl 8K (average = 1.09 × 10^7^; *p* < 0.0001) and CsCl 10K (average = 2.42 × 10^8^; *p* = 0.018). However, CsCl 8K and CsCl 10K were not significantly different from each other. Mixed samples demonstrated that a much larger loss of nucleic acid occurred earlier in the purification procedure at the PEG precipitation step as compared to individual phage samples, but with a significant further loss in the CsCl samples (*p* < 0.0001). PEG molecular weight showed significant effects only after CsCl purification with CsCl 6K (average = 5.88 × 10^8^) preserving nucleic acid better (*p* < 0.001) than either CsCl 8K (average = 3.62 × 10^8^) or CsCl 10K (average = 2.2 × 10^8^). There was only slight significance (*p* = 0.014) between CsCl 8K and CsCl 10K. As with the phage activity results, the graphs demonstrate large losses in nucleic acid labeling, but there is still a substantial amount of nucleic acid present. These losses are indicative of a change in sample intensity on the order of one or two log. For example, the ΦX CsCl 8K samples were two logs lower than the expected value (10^9^) but average fluorescent intensity was still high at 10^7^. This is demonstrated well with representative overlaid histograms (Figure 5, third panels) where the decrease in fluorescent intensity from the stock solutions can be seen giving way to an increase in lower pixel values rather than a complete loss of signal. 

### 3.4. qPCR Quantitation Confirms Loss of Phage Numbers with Processing

**Figure 6:**
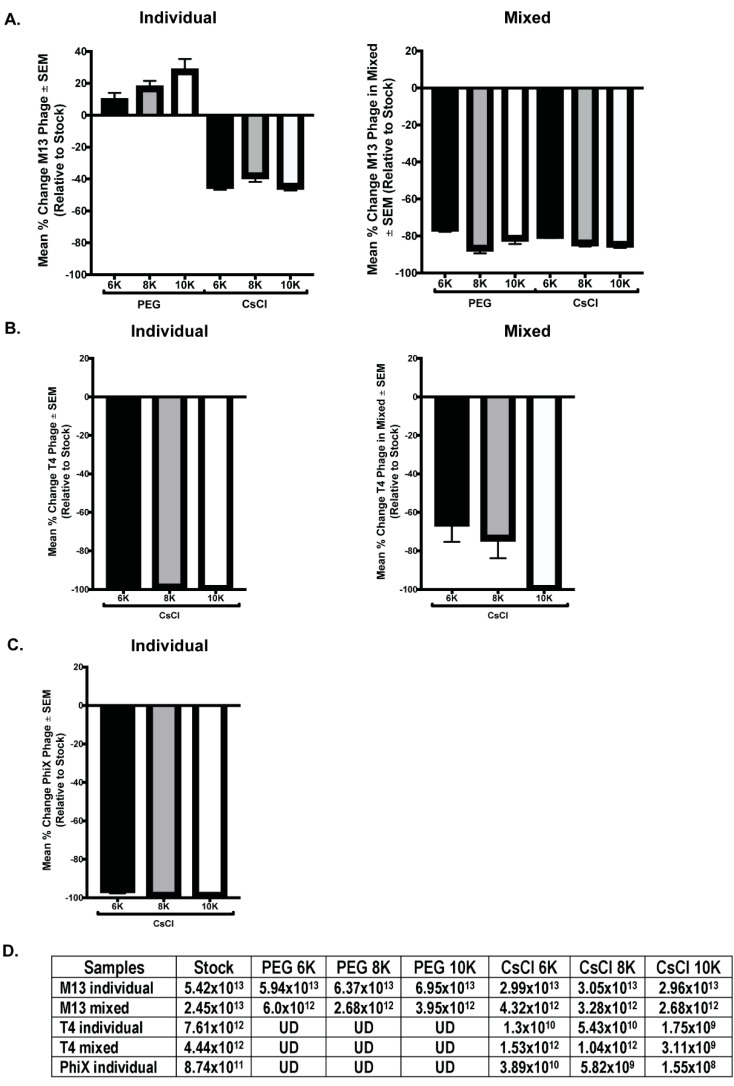
Selected phage numbers are significantly decreased after PEG precipitation and/or CsCl purification. Mean percent change in phage numbers as determined through qPCR quantitation of DNA is shown for the individual (left column) and mixed (right column) phage samples for (**A**) M13, (**B**) T4, and (**C**) ΦX. (**D**) The average numbers of phage/mL calculated for each sample set show consistent and significant decrease in phage after PEG precipitation (T4, ΦX but not M13) and CsCl purification when compared to stock. UD = undetectable.

qPCR with phage specific primers was performed on all phage samples to quantitate phage numbers. DNA was purified from 500 μL of M13 stock, individual, and mixed samples for qPCR template. T4 and ΦX samples were similarly treated, however, T4 was undetectable (UD) in all samples and ΦX was undetectable in the individual PEG and all mixed samples. Thus, a larger starting volume (2 mL) was used for DNA isolation to increase the amount of template DNA for detection. However, there was not enough volume remaining of the samples from PEG precipitation to allow for DNA isolation and qPCR quantitation (only 1 mL of these samples was collected before advancing through the purification protocol), so only the CsCl samples were processed. Despite this increase in DNA template, ΦX remained undetectable in the mixed samples by qPCR. Standard curves with purified phage DNA of known concentration were generated during each run to allow for calculation of the number of phage particles per reaction. Quantitation of the number of phages within each phage stock allowed for comparison of the expected number of phages per sample to the actual value calculated from qPCR data. As before, these results are graphically represented as a mean percentage of each phage within samples relative to the expected number of phages based on the starting stock (Figure 6). Of all phage tested, only M13 was detectable via qPCR in all purification steps, but in all cases, the number of phages detected in a sample was detrimentally affected by CsCl purification. Significant differences occurred between samples precipitated with PEG of varying molecular weight (M13 individual PEG 6K vs. PEG 10K, *p* = 0.0123; M13 mixed PEG 6K vs. PEG 8K, *p* < 0.0001; ΦX individual CsCl 6K vs. CsCl 8K, *p* = 0.0197, or CsCl 10K, *p* = 0.0248; T4 individual CsCl 8K vs. CsCl 6K, *p* = 0.002, or CsCl 10K, *p* = 0.0002; and T4 mixed CsCl 10K vs. CsCl 6K, *p* = 0.004, or CsCl 8K, *p* = 0.023). As we observed with phage activity and fluorescent intensity measures, losses as graphed do not correlate with complete loss of phage number (Figure 6D). For example, ΦX individual CsCl samples showed a mean percent change of almost -100% but the number of phage/mL calculated is still well above zero (CsCl 6K = 3.9 × 10^10^, CsCl 8K = 5.8 × 10^9^, CsCl 10K = 1.6 × 10^8^). While specific detection limits for each phage were not directly tested, qPCR results from both samples and standard curves suggest potential limits. The lowest detectable concentration of purified phage DNA used as a basis for standard curves were 0.315 ng/μL (M13), 0.125 ng/μL (T4), and 0.195 ng/μL (ΦX). Where a single bacteriophage has 3.5 × 10^−9^ ng (M13), 3.2 × 10^−7^ ng (T4), or 5.9 × 10^−8^ ng (ΦX) of nucleic acid, calculations indicate detection of the equivalent of approximately 10^7^ M13 (10^10^/mL), 10^5^ T4 (10^8^/mL), and 10^6^ ΦX (10^9^/mL) phage particles. These numbers are lower than values seen in experimental results, which show minimum detection of 10^12^ (M13), 10^9^ (T4), and 10^8^ (ΦX) phages/mL in samples by qPCR. 

## 4. Discussion

Most phage purification strategies described in the current literature were developed using a single type of phage grown in large volume on its host bacterium. These methods include a filtration step that separates phage from bacteria, concentration of phage into smaller volumes (PEG precipitation), and purification on CsCl or other types of gradients based on density differences (to eliminate contaminating factors such as proteins or bacterial debris). While effective in these simplified models, a variety of additional considerations emerge when purifying more complex phage samples such as filamentous phage display libraries or environmental phage populations. The first is heterogeneity. In phage display while the phage type remains the same, the expression of different surface peptides leads to a heterogenous population expressing low abundance and high abundance peptides [33]. In more complex environmental phage populations, heterogeneity is innate in the sizes, shapes, densities, and types of nucleic acid amongst phage constituents. In both cases, it is maintenance of the low abundance members that is challenging. Because of this, a protocol that has been optimized against a particular type of phage is likely to be less effective on a mixed population. Second, techniques that permit taxonomic identification of phages may have tradeoffs when the product is used to characterize function or phage activity. Processing a heterogenous phage population for the express purpose of isolating nucleic acid for sequencing is going to have different influences than processing for analyses of activity or function. Our purpose herein was to investigate the standard phage purification strategies of PEG precipitation and CsCl gradient purification on three representative phages of different size, shape, and density both individually and as a mixed population in order to determine the effects of each processing step on overall number and activity.

With analyses for activity (phage overlay) and number (nucleic acid fluorescence and qPCR), we can state that the most dramatic impact on phage populations occurs after CsCl purification. There are significant drops in both activity and number for all phages tested individually and in a mixed population due to CsCl gradients. While these drops are significant, samples still contain a substantial number of phages afterwards, indicating that this purification step would still be useful in scenarios where extremely pure phage is desired. That said, in instances where sequencing for population dynamics or phage activity and function are desired, this purification step is likely too detrimental to the overall sample, resulting in a biased assessment.

PEG precipitation itself provides a helpful method for concentrating large-volume samples. This simple method is certainly useful when sampling phage populations from the environment (oceanic, soil, and gastrointestinal). However, this precipitation also resulted in loss of both phage number and activity, although to a lesser extent than that seen with CsCl purification. While losses due to PEG precipitation in individual phage samples were minor in most cases, it appears that this effect is amplified when dealing with a mixed phage population. The molecular weight of the PEG used during precipitation did not seem to have a consistent trend relative to each phage tested even though there were some significant differences between samples treated with PEG of different molecular weight. Once again, these data demonstrate that this common phage purification strategy could be biasing results.

There are other considerations and phage purification strategies that exist that are beyond the scope of this work. For example, consideration of phages bound to bacterial hosts or mucin within an environmental sample prior to beginning any purification procedure. There are likely to be methods that allow for the maximal release of such phages including buffers with different salt concentrations or pH, gentle manipulation, or extended solubilization incubation. The native environmental conditions of the phage community are also a confounding factor when purifying a population. Within the gastrointestinal system, most bacterial constituents are obligate or facultative anaerobes [34,35] and, as such, the manner of collection is likely to influence their physiological state. When collecting samples from the luminal environment, maintaining anaerobicity may be required to accurately purify the population of “free” phage. Failure to maintain bacterial health during purification may result in stress-induced release of lysogenic phage [36] skewing the population and any downstream assessments. It is also possible that other described phage purification strategies, such as tangential flow filtration (TFF) [8,16] or liquid chromatography [1,17], might prove less detrimental to the characteristics of any given phage population than the methods used herein. However, these methods are more difficult and expensive, which for now, limits their use on a broader scale. Some research has been done to characterize the effects of stabilizing agents on the integrity of purified phage [37,38]. It is possible that such additives may prove beneficial to phage structure, activity, and numbers during phage purification and they offer a productive line of research, especially in the context of phage therapeutics.

Our results suggest that any type of purification strategy has disadvantages and tradeoffs and can be detrimental to overall phage numbers within a sample. This is even more true when it comes to a mixed population. Additionally, a large starting volume is required in order to obtain enough phage to become visibly detectable on a gradient or through qPCR; volumes that are not easily obtainable when dealing with small animal models for gastrointestinal phage. Furthermore, PCR and fluorescent microscopy-based detection methods for quantitation of phage are limited in their ability to define a viable, intact phage as fragments of nucleic acid are detected equally. Based on the results herein, we suggest selecting PEG specifically for the individual phage of interest (Table 1) only when concentration is necessary. The detrimental effects of CsCl should be avoided to maintain diversity and integrity as much as possible. Additionally, even though sequencing has introduced a wealth of information regarding phage constituents within the microbiome [39,40,41,42,43], it has become apparent that there is a need to move into research that investigates the activity of said phage populations in relation to health and dysbiosis. As such, it is even more important to retain not only the community structure, but the activity. Understanding how purification steps affect a variety of phage is currently the only way to optimize protocols for heterogenous phage populations as well as to develop new methods that are less invasive.

## Figures and Tables

**Table 1:** Summary of phage purification results.

Phage	Optimal Simple * Purification		Optimal Complex * Purification	Recommendation (Based on Compiled Findings)
	ACTIVITY	NUMBER	NUMBER	
M13	PEG6K	PEG10K	PEG6K	If using phage for activity or attempting to maintain within a complex population, use PEG6K when concentration is necessary. If isolating for highest numbers, use PEG10K. Avoid CsCl.
T4	PEG6K	PEG6K	PEG8K ^†^	If concentration is necessary for analysis of phage activity or to maintain highest numbers from bacterial culture, use PEG6K. If isolating for highest numbers in complex phage population, use PEG8K. Avoid CsCl.
ΦX	PEG6K	PEG6/8/10K	PEG8K ^†^	If concentration is necessary during phage purification from bacterial culture for activity, use PEG6K. If isolating for highest numbers in complex phage population, use PEG8K. Any PEG can be used to retain numbers in purifications from bacterial culture. Avoid CsCl.

* Simple = single phage grown in bacterial culture; Complex = mixed phage population from environmental source; ^†^ Based solely on non-specific fluorescent microscopy counts.
